# Association between plant-based diets and depression in older adults with heart disease: the mediating role of sleep disturbances

**DOI:** 10.3389/fnut.2025.1567436

**Published:** 2025-04-16

**Authors:** Yaqun Yu, Yueying Cheng, Nan Cheng, Jie Zhang, Qitao Xu, Yawen Wang, Wei Zhou, Chao Yan, Huiqiong Li, Zhiyun Gong

**Affiliations:** Department of Cardiac Vascular Surgery, The First Medical Center of the People's Liberation Army General Hospital, Beijing, China

**Keywords:** heart disease, depression, plant-based diets, cross-sectional study, sleep, older adults

## Abstract

**Background:**

Depression is not uncommon among older adults with heart diseases and is related to poor prognosis at clinical setting. We aim to explore the association between plant-based diets and depression in older adults with heart disease and further investigate the mediating role of sleep disturbances in this relationship.

**Methods:**

A cross-sectional sample of 2039 older adults with heart diseases were recruited from the 2018 Chinese Longitudinal Healthy Longevity Survey (CLHLS). Each individual completed assessments on dietary frequency, depression, sleep quality and duration. Plant-based diet index (PDI), healthy plant-based diet index (hPDI) and unhealthy plant-based diet index (uPDI) were calculated. Logistic regression models and restricted cubic spline curves (RCS) were employed to explore the relationship between plant-based diets and depression in older adults with heart disease. Meanwhile, mediation analysis was used to investigate the mediating roles of sleep quality and sleep duration.

**Results:**

The higher the PDI (OR: 0.56, 95% CI: 0.36–0.88) and the hPDI (OR: 0.39, 95% CI: 0.24–0.62), the lower the risk of depression in older adults with heart disease. Conversely, the higher the uPDI, the higher the risk of depression (OR: 1.76, 95% CI: 1.07–2.92). RCS further confirmed a negative linear dose–response relationship between PDI, hPDI and depression in older adults with heart disease, and a positive trend was found between uPDI and depression. Notably, sleep quality (Indirect effect: −0.031, mediated proportion: 61%) partially mediated the relationship between PDI and depression. In the sex-based subgroup analysis, uPDI was only associated with a higher risk of depression in females.

**Conclusion:**

This is the first study to suggest a significant negative relationship between plant-based diets and depression in older adults with heart disease. Sleep quality plays a mediating role in the association between plant-based diets and depression. Optimizing the dietary structure and improve sleep quality may help reduce the risk of depression in older adults with heart disease.

## 1 Introduction

With the aging of global population, the prevalence of cardiovascular diseases (CVD) among the elderly is showing a sustained upward trend (1). According to Global Burden of Cardiovascular Diseases 1999–2022, the proportion of deaths caused by CVD among the elderly accounts for as high as 66% of the total deaths (2). The prevalence of depression among older adults with CVD is two to three times higher than that in the general population (3, 4), especially among patients who have had myocardial infarction, heart failure, and coronary artery bypass grafting (5, 6). Among older adults with coronary artery disease (CAD), approximately 30% experience an exacerbation of depressive symptoms, and 15–20% are diagnosed with major depressive disorder (MDD). A large body of studies have shown a significant association between depression and CVD, and depression is positively correlated with the incidence and mortality of heart diseases, including increased mortality and readmission rates (6–8). After adjusted for traditional risk factors of heart disease, the impact of depression on the poor prognosis of CVD still remains significant (9). Risk factors associated with depression in older adults with CVD are multi-faceted, involving various fields such as psychological, social, and biological (10–13). Preventing the onset of depression is of significant importance in aspects such as improving the prognosis of older adults with heart disease, reducing medical costs, and enhancing the patients’ quality of life (14, 15).

Dietary patterns in older adults with heart disease may serve as a strategy for depression prevention, since dietary factors play a crucial role in the development of depression (16, 17). A plant-based diet may affect mood by altering the intake of neurotransmitters such as tryptophan (18). Some studies suggest that a healthy diet pattern can reduce the risk of depression, while an unhealthy one may increase it (19, 20). For older adults with heart disease, dietary factors may be of even greater importance since dietary factors are not only related to depression but also closely associated with the development of CVD (21–23). Plant-based diets refer to a dietary pattern that emphasizes plant-based foods as the main source of food (24). Satija et al. used a hierarchical approach to create three plant-based diet indices: the overall Plant-based Diet Index (PDI), the unhealthy Plant-based Diet Index (uPDI), and the healthy Plant-based Diet Index (hPDI) (25). This model takes into account the intake of various plant-based and animal-based foods. It assigns different scores according to their impacts on health to evaluate the dietary pattern. a large number of studies suggested that PDI is associated with a lower risk of stroke (26), diabetes (27) CVD, and overall mortality (28). A plant-based diet is typically rich in antioxidants, such as vitamin C, folic acid, and phytochemicals, which play a critical role in reducing neuro-oxidative damage and protecting neurons (29). A cross-sectional study of multi-center data indicated that no significant association between PDI, hPDI and depression or anxiety exists, while uPDI is associated with an increased risk of depression and anxiety (30). Other studies on different populations, including the healthy middle-aged and the elderly, adolescents, and patients with diabetes, found that adhering to hPDI dietary pattern is associated with a reduced risk of dementia and depression, whereas adhering to uPDI dietary pattern is associated with an increased risk (31–33). However, the relationship between the plant-based diets and depression in older adults with heart diseases has not been explored yet.

Sleep disturbances are prevalent among the elderly population, particularly among those with heart diseases (34). The relationship between sleep disturbances and depression has been reported by numerous studies (35, 36). Some regarded sleep abnormalities as common symptoms of depression, but a bidirectional relationship between sleep and depression has also been found (37, 38). A longitudinal study suggest that sleep problems often indicate the potential emergence of depression (39). Meanwhile, patients with depression also tend to have sleep issues (40). On the other hand, a healthy plant-based diet can improve sleep quality, while insufficient intake of plant-based diet may trigger sleep problems (41–43). However, the role of sleep disturbances in the relationship between PDI and depression among older adults with heart disease remains to be explored.

To fill the gaps in the aforementioned research, we aim to explore the relationship between plant-based diets and depression in older adults with heart disease using a large, nationally representative population sample. Meanwhile, the mediating role of disturbances in sleep quality and duration in the association between plant-based diets and depression. Our work has significant clinical implications as it may indicate an evidence-based approach for the prevention of depression in older adults with heart disease.

## 2 Materials and methods

### 2.1 Research subjects

The participants in our study are recruited from The Chinese Longitudinal Healthy Longevity Survey (CLHLS) (44). This project is a follow-up survey of the elderly organized by the Center for Healthy Aging and Development Studies/National School of Development at Peking University. It began in 1998 and were carried out periodically, with data collection conducted every 3 to 4 years for the same population. The survey covers 23 provinces, municipalities, and autonomous regions across the country. The questionnaires are divided into two types: the questionnaire for surviving interviewees and the questionnaire for family members of deceased elderly people. The survey subjects include the elderly aged 65 and above and adult children aged 35–64. A total of 15,874 elderly people over 65 were interviewed, and information on 2,226 elderly people who died during the period from 2014 to 2018 was collected. This survey has obtained formal authorization from the Biomedical Ethics Committee of Peking University, China (Approval No.: IRB00001052-13074), and every interviewee participating in the survey has signed an informed consent form as required. Studies using data from CLHLS datasets have been previously published (45–47).

We utilized the questionnaire data of surviving interviewees from the CLHLS 2018 (Total participants:15874). Participants aged below 65 years old and with missing values in assessment of depression, sleep, and dietary intake were excluded. Eventually, 2039 older adults with heart diseases were included in the analysis (see Supplementary Figure S1).

### 2.2 Outcome measures

In this study, the dependent variable is the presence of depression. A shortened version of Center for Epidemiological Studies Depression Scale (CES-D-9) to assess depression, by using 9 items excluding sleep assessment (48, 49). The CES-D-9 adopts a 4-point Likert scale. Responses to each question are “rarely,” “occasionally,” “often,” and “most of the time.” Scores for items with positive effects are reversed. The total score of CES-D-9 ranges from 0 to 27. We defined a CES-D-9 ≥ 9 as indicating the presence of depression. The Cronbach’sαof CES-D-9 is 0.77 in our study, demonstrating good internal consistency and reliability.

### 2.3 Measurement of PDI

Based on previous research (25, 43, 50), we evaluated plant-based diets by calculation of the PDI (Plant-based Diet Index), healthy plant-based diet index (hPDI) and unhealthy plant-based diet index (uPDI) based on participants’ dietary information using a simplified food frequency questionnaire, which was tested for reliability and validity in previous studies (51, 52). Taking into account the composition of various foods, 16 of the most commonly consumed foods were classified into three groups: animal-sourced foods, healthy plant-based foods, and unhealthy plant-based foods. For the 16 food groups, a score rating from 1–5 was assigned. Regarding PDI, the scores of plant-based foods increase from 1 to 5 as the consumption frequency rises, while the scores of animal-based foods show a reversed trend. hPDI assigns positive scores to healthy plant-based foods and negative scores to unhealthy plant-based foods as well as animal-based foods. In contrast, uPDI gives positive scores to unhealthy plant-based foods and negative scores to healthy plant-based foods and animal-based foods. Then, all the scores are summed up to obtain the final scores of PDI, hPDI, and uPDI for the 16 food groups. For more detailed information on the construction of PDI, hPDI, and uPDI, please refer to Supplementary Table S1. Theoretically, the score range of these indices varies from 16 to 80 points. A higher score of PDI and hPDI indicates a stronger compliance with plant-based diets, while a higher uPDI indicates the opposite. In addition, we divided the scores of PDI, hPDI, and uPDI into four quartiles, Q1, Q2, Q3, and Q4, for subsequent analysis. In our study, the Cronbach’sαof PDI, hPDI and uPDI were 0.79, 0.76 and 0.77, respectively.

### 2.4 Assessment of sleep health

The assessment of sleep health is defined by two components: sleep quality and sleep duration. We rely on two questions in the questionnaire for evaluation. One is “How is your current sleep quality?” and the other is “How many hours do you usually sleep per day currently?.” In terms of sleep quality, for the responses of “very good,” “good,” “fair,” “poor,” and “very poor” from the respondents, they are assigned scores from 1 to 5 in sequence. The higher the score, the worse the sleep quality. Regarding sleep duration, based on the sleep-time recommendations for cardiovascular disease patients in multiple studies (34, 53), the sleep duration is classified into three categories: less than 6 h, 6–8 h, and more than 8 h. Among these, a sleep duration of less than 6 h or more than 8 h is assigned a value of “1” during the assessment, indicating disturbance of sleep duration, while the normal sleep duration (6–8 h) is assigned a value of “0,” suggesting no disturbances in sleep duration.

### 2.5 Covariates

Based on previous relevant studies (54–58), the covariates involved in this study cover two aspects: the demographic and socioeconomic characteristics, including gender, age, and place of residence, marital status, educational status, occupation, annual family income, housing property, and regional factors (from south or north China); the life style and comorbidity: including factors like smoking, alcohol consumption, exercise, body mass index, diabetes, dyslipidemia, and cerebrovascular diseases. Additionally, we have created and included Directed Acyclic Graphs (DAG) diagrams to visually represent the relationships between the variables studied (see Supplementary Figure S2).

### 2.6 Statistical analysis

Categorical variables were described using frequencies and percentages. Since none of the numerical variables passed the normality test, continuous variables were described using the median (interquartile range). The Chi-square test and Mann–Whitney U test were used to compare the differences between depressed and non-depressed individuals for categorical variables and continuous variables, respectively. Three logistic regression models were used to systematically examine the relationship between plant-based diets and depression while controlling for potential confounders at different levels. Model A did not adjust for any covariates to identify the raw association and lays the groundwork for further analysis (Crude model). Model B was adjusted for key demographic and socioeconomic variables including gender, age, place of residence, geographical region, marital status, education level, occupation, annual family income, and housing property. Model C was further adjusted for lifestyle and comorbidity factors such as smoking, alcohol consumption, exercise, BMI, diabetes, cerebrovascular diseases, and dyslipidemia based on Model B. Restricted cubic spline (RCS) with four knots was used to explore the dose–response relationships between PDI, uPDI, hPDI and depression in older adults with heart disease. In addition, subgroup analyses by different genders were conducted. Subsequently, Spearman correlation analysis was used to explore the correlations among PDI, uPDI, hPDI, sleep quality, sleep duration, and depression scores. Then, the mediating effects of sleep quality and sleep duration disorders in the relationship between plant-based diets and depression were further tested separately. The Bootstrap method was used to evaluate their mediating effects and 95% CI. PDI, uPDI, and hPDI were, respectively, included as independent variables in three mediating models. All statistical analyses were performed using R (version 4.4.1). The R package “rms” was used for restricted cubic spline (RCS) analysis, and the “mediation” package was used for mediation analysis. A two-sided *p*-value<0.05 was defined as significant in all tests.

## 3 Results

### 3.1 Characteristics of study participants

In this study, 2039 older adults with heart disease were included, 827 (40.6%) were male and 1,212 (59.4%) were female. The median age of these participants was 82.8 (IQR, 75.0–90.0) years old. There were 848 (41.6%) urban residents, 1,112 (54.8%) widowed individuals, and 1,042 (58.0%) participants living in the southern region of China. The specific baseline characteristics grouped by the presence or absence of depression are presented in Table 1.

**Table 1:** Characteristics of older adults with heart disease.

Characteristics	Overall (*n* = 2039)	Depression (*n* = 377)	Non-depression (*n* = 1,662)	*p*-value
Gender, *n* (%)				<0.001
Male	827 (40.6)	121 (32.1)	706 (42.5)	
Female	1,212 (59.4)	256 (67.9)	956 (57.5)	
Age, years, median [IQR]	82.8[75.0,90.0]	83.0[76.0,89.0]	82.7[75.0,90.0]	0.574
Age group, *n* (%)				0.108
<75	467 (22.9)	74 (19.6)	393 (23.6)	
> = 75	1,572 (77.1)	303 (80.4)	1,269 (76.4)	
Residence, *n* (%)				0.668
Urban	848 (41.6)	161 (42.7)	687 (41.3)	
Rural	1,191 (58.4)	216 (57.3)	975 (58.7)	
Geographic region, *n* (%)				<0.001
North China	756 (42.0)	102 (30.5)	654 (44.7)	
South China	1,042(58.0)	232 (69.5)	810 (55.3)	
Marital status, *n* (%)				<0.001
Married/cohabitating	916 (45.2)	140 (37.2)	776 (47.0)	
Widowed	1,112 (54.8)	236 (62.8)	876 (53.0)	
With formal education, *n* (%)	280 (15.6)	55 (16.8)	225 (15.3)	0.568
Occupation, *n* (%)				0.049
Professional and technical personnel	244 (13.6)	34 (10.3)	210 (14.3)	
Governmental, institutional, or managerial personnel	150 (8.3)	21 (6.4)	129 (8.8)	
General staff, service personnel or workers	474 (26.4)	104 (31.6)	370 (25.2)	
Farmers	759 (42.2)	136 (41.3)	623 (42.4)	
Other	171 (9.5)	34 (10.3)	137 (9.3)	
Annual household income, *n* (%)				0.002
<50,000	1,067 (53.9)	205 (56.6)	862 (53.2)	
50,000 ~ 80,000	260 (13.1)	62 (17.1)	198 (12.2)	
>80,000	654 (33.0)	95 (26.2)	559 (34.5)	
Housing nature, *n* (%)				0.760
Purchased or self-built	1,569 (81.6)	279 (80.9)	1,290 (81.7)	
Other	354 (18.4)	66 (19.1)	288 (18.3)	
Smoking, *n* (%)				0.028
Never	1,459 (71.8)	291 (77.4)	1,168 (70.6)	
Former	341 (16.8)	49 (13.0)	292 (17.6)	
Current	231 (11.4)	36 (9.6)	195 (11.8)	
Drinking alcohol, *n* (%)				0.191
Never	1,541 (76.4)	297 (79.6)	1,244 (75.7)	
Former	265 (13.1)	46 (12.3)	219 (13.3)	
Current	210 (10.4)	30 (8.0)	180 (11.0)	
Exercise, *n* (%)				<0.001
Never	1,030 (51.0)	248 (66.5)	782 (47.5)	
Former	223 (11.1)	45 (12.1)	178 (10.8)	
Current	756 (37.9)	80 (21.4)	685 (41.6)	
BMI (kg/m^2^), median [IQR]	23.2[20.6,26.0]	23.0[20.0,25.5]	24.3[20.7,26.0]	0.004
Diabetes, *n* (%)	411 (21.8)	102 (30.0)	309 (20.0)	<0.001
Cerebrovascular disease, *n* (%)	456 (24.2)	119 (34.5)	337 (21.9)	<0.001
Dyslipidemia, *n* (%)	286 (15.5)	69 (20.6)	217 (14.4)	0.006
PDI, median [IQR]	48.9 [45.0, 52.0]	48.0 [44.0, 51.0]	49.1 [46.0, 53.0]	<0.001
hPDI, median [IQR]	47.5 [44.0, 51.0]	46.3 [43.0, 49.0]	47.7 [44.0, 51.0]	<0.001
uPDI, median [IQR]	49.2 [44.0, 55.0]	51.3 [45.0, 57.0]	48.8 [43.0, 54.0]	<0.001
Sleep quality, median [IQR]	2.6 [2.0, 3.0]	3.2 [3.0,4.0]	2.5 [2.0, 3.0]	<0.001
Sleep duration(hours/day), median [IQR]	7.0 [6.0, 8.0]	6.3 [5.0, 7.0]	7.2 [6.0, 8.0]	<0.001

IQR, inter quartile range; BMI, body mass index; PDI, plant-based diet index; uPDI, unhealthy plant-based diet index; hPDI, healthy plant-based diet index.

### 3.2 Association between plant-based diets and depression

In Model A, PDI (OR: 0.52, 95% CI: 0.37–0.70) and hPDI (OR: 0.44, 95% CI: 0.31–0.62) were associated with lower risk of depression. However, uPDI was associated with higher prevalence of depression (OR: 2.05, 95% CI: 1.49–2.82).

In the partially adjusted model (Model B), PDI (OR: 0.52, 95% CI: 0.35–0.77) and hPDI (OR: 0.45, 95% CI: 0.30–0.67) were still associated with lower risk of depression, while uPDI showed a positive connection with depression (OR: 1.95, 95% CI: 1.27–3.01).

In the fully adjusted model (Model C), greater adherence to PDI (OR: 0.56, 95% CI: 0.36–0.88) and hPDI (OR: 0.39, 95% CI: 0.24–0.62) were associated with a lower prevalence of depression in older adults with heart disease, while uPDI still positively associated with risk of depression (OR: 1.76, 95% CI: 1.07–2.92) (see Table 2).

**Table 2:** Associations between PDI and risks of depressive symptoms among older adults with heart disease.^a^

	Model A			Model B			Model C		
	OR	95% CI	*p-*value	OR	95% CI	*p*-value	OR	95% CI	*p*-value
PDI									
Continuous	0.96	0.94–0.98	<0.001	0.96	0.94–0.99	0.004	0.97	0.95–1.00	0.083
Q1	Reference			Reference			Reference		
Q2	0.82	0.59–1.11	0.200	0.80	0.55–1.17	0.254	0.70	0.45–1.10	0.122
Q3	0.70	0.51–0.97	0.032	0.78	0.53–1.15	0.203	0.78	0.50–1.22	0.269
Q4	0.52	0.37–0.70	<0.001	0.52	0.35–0.77	0.001	0.56	0.36–0.88	0.011
*p*-trend			<0.001			0.002			0.034
uPDI									
Continuous	1.04	1.03–1.06	<0.001	1.05	1.03–1.07	<0.001	1.04	1.02–1.07	<0.001
Q1	Reference			Reference			Reference		
Q2	1.08	0.75–1.54	0.668	0.98	0.65–1.49	0.927	0.89	0.55–1.43	0.621
Q3	1.36	0.98–1.90	0.067	1.14	0.75–1.73	0.547	0.81	0.50–1.33	0.410
Q4	2.05	1.49–2.82	<0.001	1.95	1.27–3.01	0.003	1.76	1.07–2.92	0.026
*p*-trend			<0.001			0.002			0.035
hPDI									
Continuous	0.95	0.93–0.97	<0.001	0.95	0.93–0.98	<0.001	0.94	0.92–0.98	<0.001
Q1	Reference			Reference			Reference		
Q2	0.85	0.62–1.17	0.324	0.71	0.48–1.04	0.082	0.68	0.43–1.05	0.085
Q3	0.80	0.60–1.09	0.153	0.80	0.57–1.13	0.205	0.73	0.49–1.08	0.117
Q4	0.44	0.31–0.62	<0.001	0.45	0.30–0.67	<0.001	0.39	0.24–0.62	<0.001
*p*-trend			<0.001			<0.001			<0.001

CI, confidence interval; OR, odds ratio. Model A did not adjust for any covariates. Model B adjusted for gender, age, residence, geographic region, marital status, education, occupation, annual household income and housing nature. Model C further adjusted for smoking, drinking alcohol, exercise, BMI, diabetes, cerebrovascular disease, dyslipidemia based on Model B.

a The associations between PDI and the risks of Depressive symptoms are presented as ORs (95% CI).

**Figure 1:**
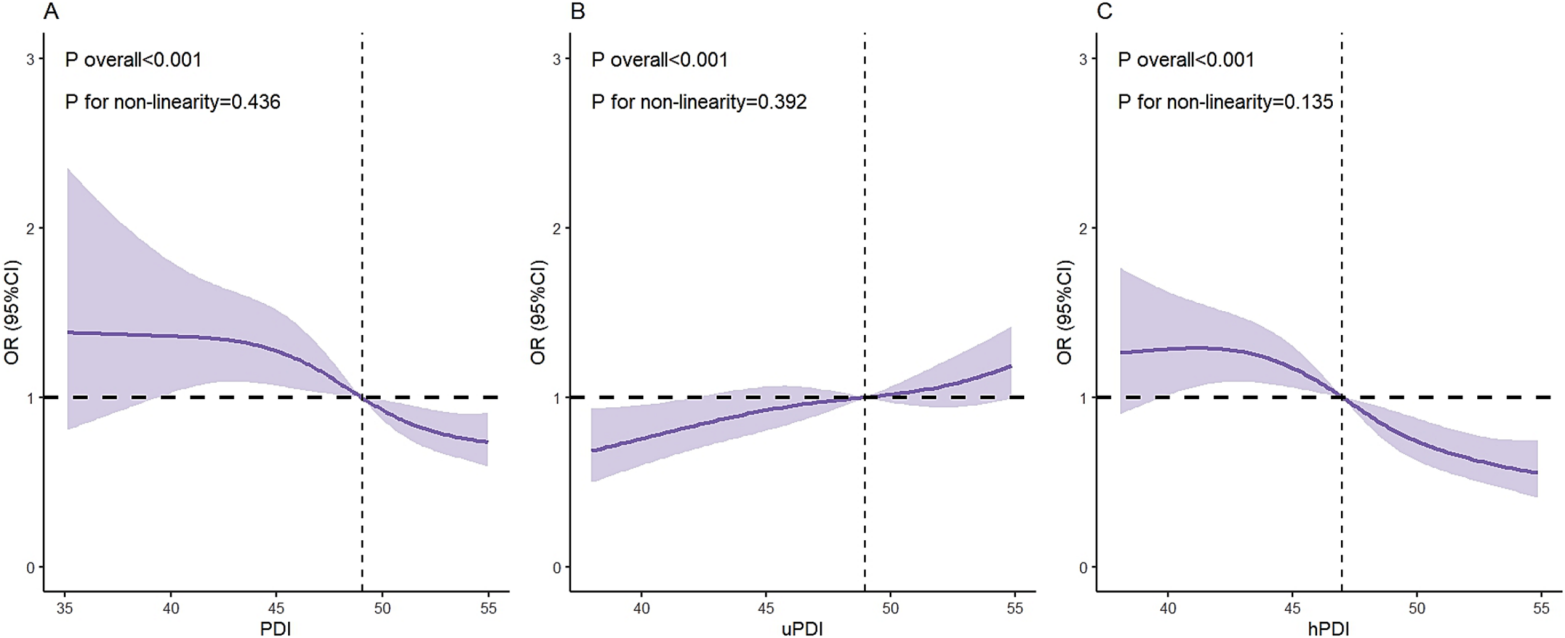
Results of restricted cubic spline analysis. **(A)** PDI; **(B)** uPDI; **(C)** hPDI.

The results of the RCS curves indicated that both the PDI and hPDI diet showed a negative linear dose–response relationship with depression in older adults with heart disease, while the uPDI diet was positively correlated with depression in these patients (see Figure 1).

### 3.3 Subgroup analysis

The subgroup analysis showed that, for the uPDI, compared with the female patients in the lowest quartile (Q1), highest quartile (Q4) was associated with an increased risk of depression (OR: 1.97, 95% CI: 1.04–3.82). For the hPDI, compared with the lowest quartile, Q4 in both the male (OR: 0.26, 95% CI: 0.11–0.58) and female (OR: 0.49, 95% CI: 0.26–0.91) subgroups had a lower risk of depression (Table 3).

**Table 3:** Sex differences in the associations between PDI and Risks of Depressive Symptoms among older adults with heart disease.^a^

	Male			Female		
	OR	95% CI	*p*-value	OR	95% CI	*P*-VALUE
PDI						
Continuous	0.97	0.92–1.03	0.302	0.97	0.93–1.01	0.134
Q1	Reference			Reference		
Q2	0.51	0.22–1.16	0.106	0.80	0.46–1.39	0.425
Q3	0.84	0.38–1.86	0.661	0.63	0.35–1.11	0.108
Q4	0.50	0.23–1.12	0.088	0.56	0.31–1.00	0.048
*p*-trend			0.283			0.045
uPDI						
Continuous	1.04	1.00–1.08	0.076	1.05	1.02–1.08	0.001
Q1	Reference			Reference		
Q2	0.75	0.35–1.59	0.467	1.03	0.53–1.99	0.936
Q3	0.93	0.41–2.10	0.868	0.86	0.45–1.66	0.653
Q4	1.75	0.73–4.20	0.211	1.97	1.04–3.82	0.040
*p*-trend			0.283			0.045
hPDI						
Continuous	0.91	0.87–0.97	0.003	0.96	0.92–1.00	0.035
Q1	Reference			Reference		
Q2	0.55	0.25–1.21	0.142	0.75	0.42–1.31	0.312
Q3	0.60	0.30–1.20	0.151	0.81	0.49–1.34	0.408
Q4	0.26	0.11–0.58	0.001	0.49	0.26–0.91	0.025
*p*-trend			0.003			0.055

CI, confidence interval; OR, odds ratio. Model adjusted for age, residence, geographic region, marital status, education, occupation, annual household income, housing nature, smoking, drinking alcohol, exercise, BMI, diabetes, cerebrovascular disease, dyslipidemia.

a The associations between PDI and the risks of Depressive symptoms are presented as ORs (95% CI).

### 3.4 Spearman correlation analysis

**Figure 2:**
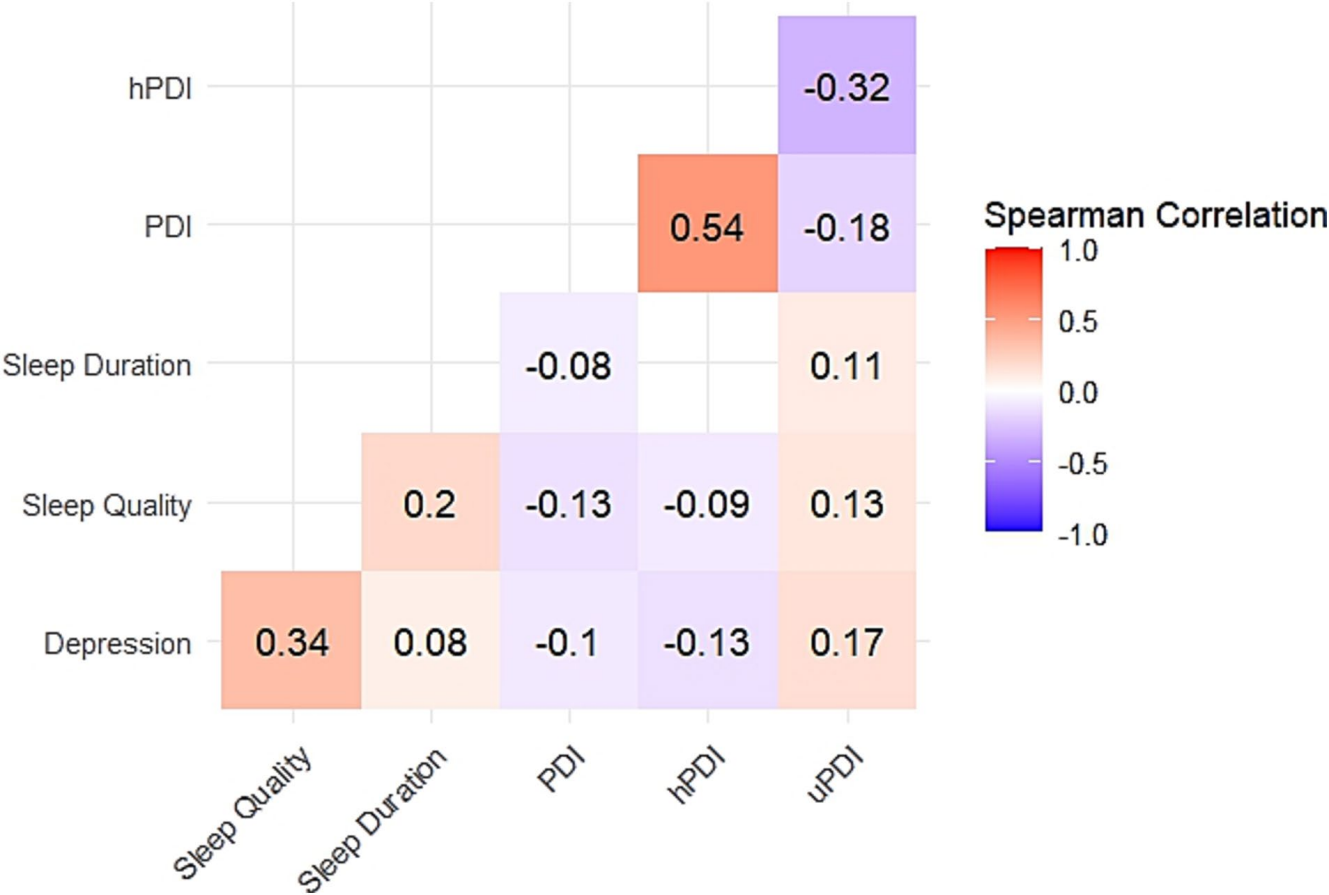
Results of Spearman correlation analysis. Red represents positive correlation, blue represents negative correlation, and the darker the color, the stronger the correlation. White indicates that there is no significant correlation between variables. All *p* < 0.001 for correlation with color.

Depressive symptoms in heart disease patients were positively correlated with poor sleep quality (r = 0.34) and sleep duration disturbances (r = 0.08). Depressive symptoms in older adults with heart disease showed a negative correlation with the PDI (r = −0.10) and the hPDI (r = −0.13), and a positive correlation with the uPDI (r = 0.17). Poor sleep quality was negatively correlated with the PDI (r = −0.13), and hPDI (r = −0.09) and positively correlated with the uPDI (r = 0.13), all *p*-values<0.001 (Figure 2).

### 3.5 The mediating role of sleep quality and sleep duration

**Figure 3:**
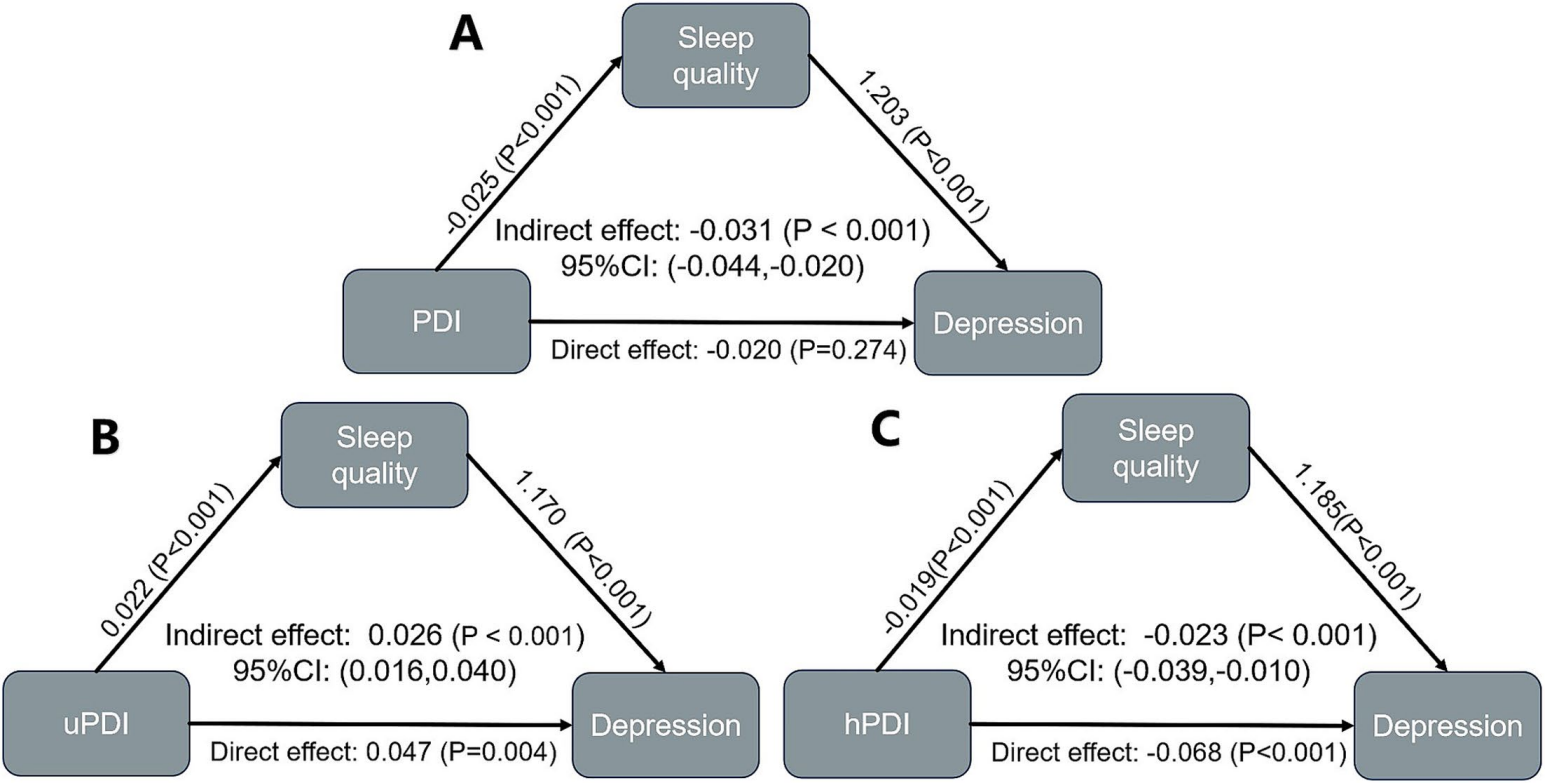
Results of mediation analysis for sleep quality. **(A)** PDI; **(B)** uPDI; **(C)** hPDI.

Sleep quality has a significant mediating effect in the relationship between PDI and depression in older adults with heart disease (Indirect effect: −0.031, 95% CI: −0.044 to −0.020). Sleep quality also has a significant mediating effect in the relationship between uPDI and depression (Indirect effect: 0.026, 95% CI: 0.016 to 0.040). Similarly, a mediating effect exists between hPDI and depression (Indirect effect: −0.023, 95% CI: −0.039 to −0.010) (Figure 3, Supplementary Table S2). The mediating effect of sleep duration in the relationship between plant-based diets and depression in older adults with heart disease was not significant (Supplementary Figure S3).

## 4 Discussion

For the first time, our study deeply explored the association between plant-based diets and depression in older adults with heart disease and further analyzed the mediating role played by sleep disturbances. We found a significant negative relationship between plant-based diets and depression. In addition, improved sleep quality showed a mediating effect in the association between healthy plant-based diets and depression. Sex difference was also examined and significant relationship between uPDI and the occurrence of depression was found in women, while there was no such relationship in men.

Participants with higher levels of PDI and hPDI had lower prevalence of depression, which may indicate that the higher the patients’ compliance with a healthy plant-based diet, the lower the risk of developing depressive symptoms. Multiple studies, such as those on young women (59) and the elderly (60), have shown that a high-quality plant-based diet is associated with a reduced risk of depressive symptoms. A cross-sectional study by Lee et al. found that lifestyle changes such as diet might have a positive impact on depression (61). Building on the above-mentioned studies, this research also confirmed the negative correlation between plant-based diets and depression among heart disease patients, further enriching the research in this field. Previous studies have shown that plant-based diets are rich in dietary fiber, which can improve the gut microbiota, and gut microbiota dysbiosis is associated with the occurrence of depression (62, 63). Moreover, healthy plant-based diets are abundant in unsaturated fatty acids and antioxidant components like flavonoids and vitamins. These substances have been found to possess anti-inflammatory properties (64, 65). Inflammation, on the other hand, is of great significance in the pathophysiology of both heart disease and depression (66–68).

Disturbances in sleep quality and sleep duration are another major risk factors for depression in older adults with heart disease. Of note, the correlation between sleep quality and depression seemed stronger, while the relationship between sleep duration and depression is relatively weaker. Some studies have shown that sleep disorders are important risk factors for depression and have a bidirectional association with depression (39, 69). Sleep disorders are associated with more severe depressive outcomes, including suicide risk, comorbidity with anxiety disorders, and others (70, 71). Some studies on the mechanisms of sleep and the onset of depression have found that sleep disturbances may disrupt the function of neural synaptic networks, including those in the hippocampus and prefrontal cortex, leading to impaired cognitive function. This process is associated with the development of depression (72, 73). In addition, patients with depression often exhibit excessive arousal. Depressive symptoms and sleep disturbances may interact with each other through common neurobiological pathways (74, 75).

Another significant finding of this study is that plant-based diets may improve the sleep quality and thus help lower the risk of depression in older adults with heart disease. Specifically, a healthy plant-based diet pattern helps optimize sleep quality, thus playing a role in reducing the risk of depression. In contrast, an unhealthy plant-based diet pattern may have a negative impact on sleep quality, thereby contributing to the occurrence or exacerbation of depression. Previous studies have found that adherence to a healthy plant-based diet is positively correlated with optimal sleep quality (42), and improved sleep can reduce the risk of depression (76, 77). From a biological perspective, phytoestrogens such as flavonoids, isoflavones, and lignans contained in plant-based diets can improve sleep quality. Moreover, a variety of plant-based foods such as fruits and vegetables can enhance sleep health by increasing the levels of melatonin and serotonin (78). Improved sleep can affect the regulatory mechanisms of the nervous system, including the circadian rhythm, neurotransmitter systems, and melatonin, thus alleviating symptoms of depression (79), the above-mentioned studies all support the findings of this research.

In addition, through sex-based subgroup analysis, this study further found that the uPDI diet was associated with the risk of depression in the female subgroup but not in males. As for the hPDI diet pattern, the protective effect of plant-based diets on depression in female older adults with heart disease was relatively weak. Such sex differences are not uncommon in research in the field of mental health. According to the theory of psychological gender differences, men and women differ in many aspects such as physiological structure, hormone levels, and social roles. These factors cause them to have different psychological and physiological responses to the same external stimuli (such as changes in diet patterns) (80). A large number of previous studies have found that women are more prone to be troubled by depression (81, 82), which is consistent with the conclusion of this study.

This study has several advantages. We used a large, nationally representative sample population, taking the lead in exploring the association between plant-based diets and depression in Chinese older adults with heart disease, and deeply analyzing the mediating role of sleep disorders in this relationship. When analyzing the relationship between PDI and depression in older adults with heart disease, this study excluded a variety of confounding factors to ensure the robustness of the results.

This study also has several limitations. Firstly, the cross-sectional design adopted in this study limit the ability to establish a causal relationship. The findings of this study need to be confirmed by future longitudinal intervention-based studies. Secondly, the data on diet, depression, and sleep status in this study were all derived from patients’ self-reports. Participants may have recall bias, leading to inaccurate assessments of some participants. Future research needs to adopt more objective assessment methods to explore this complex relationship more comprehensively, in-depth, and accurately, providing a more reliable basis for the health management of older adults with heart disease.

## 5 Conclusion

In conclusion, plant-based diets are associated with a lower risk depression in Chinese older adults with heart disease, and sleep quality disturbances can partially mediate this relationship. The results of this study provide a clear and evidence-based direction for dietary guidance practices for depression prevention in older adults with heart disease. By optimizing the diet structure and actively improving patients’ sleep quality, the risk of depression in older adults with heart disease could possibly reduce, as well as the prognosis of CVD.

## Data Availability

Publicly available datasets were analyzed in this study. This data can be found here: the data supporting the results of this study are publicly available in the China Longitudinal Healthy Longevity Survey (CLHLS) on the Peking University Open Data Platform: https://opendata.pku.edu.cn/dataverse/CHADS.
