# Supplementary material for: Association between plant-based diets and depression in older adults with heart disease: the mediating role of sleep disturbances
Source: Front Nutr. 2025 Apr 16;12:1567436. doi: 10.3389/fnut.2025.1567436 (PMC12040692; doi:10.3389/fnut.2025.1567436)
Supplement: Supplementary file 1 [file Table_1.DOCX]

| **Supplementary Table S1. Plant-based diet index scoring** | | | | | |
| --- | --- | --- | --- | --- | --- |
| Food | Food | Frequency | PDI score | hPDI score | uPDI score |
| Plant food | Whole grain | Yes | 5 | 5 | 1 |
|  |  | No | 1 | 1 | 5 |
|  | Refined grain | Yes | 5 | 1 | 5 |
|  |  | No | 1 | 5 | 1 |
|  | Vegetable oil | Yes | 5 | 5 | 1 |
|  |  | No | 1 | 1 | 5 |
|  | Fresh vegetable | Almost everyday | 5 | 5 | 1 |
|  |  | Quite often | 4 | 4 | 2 |
|  |  | Occasionally | 2 | 2 | 4 |
|  |  | Rarely or never | 1 | 1 | 5 |
|  | Fresh fruit | Almost everyday | 5 | 5 | 1 |
|  |  | Quite often | 4 | 4 | 2 |
|  |  | Occasionally | 2 | 2 | 4 |
|  |  | Rarely or never | 1 | 1 | 5 |
|  | Garlic | Almost everyday | 5 | 5 | 1 |
|  |  | ≥1 time/week | 4 | 4 | 2 |
|  |  | ≥1 time/month | 3 | 3 | 3 |
|  |  | Occasionally | 2 | 2 | 4 |
|  |  | Rarely or never | 1 | 1 | 5 |
|  | Legume | Almost everyday | 5 | 5 | 1 |
|  |  | ≥1 time/week | 4 | 4 | 2 |
|  |  | ≥1 time/month | 3 | 3 | 3 |
|  |  | Occasionally | 2 | 2 | 4 |
|  |  | Rarely or never | 1 | 1 | 5 |
|  | Nut | Almost everyday | 5 | 5 | 1 |
|  |  | ≥1 time/week | 4 | 4 | 2 |
|  |  | ≥1 time/month | 3 | 3 | 3 |
|  |  | Occasionally | 2 | 2 | 4 |
|  |  | Rarely or never | 1 | 1 | 5 |
|  | Tea | Almost everyday | 5 | 5 | 1 |
|  |  | ≥1 time/week | 4 | 4 | 2 |
|  |  | ≥1 time/month | 3 | 3 | 3 |
|  |  | Occasionally | 2 | 2 | 4 |
|  |  | Rarely or never | 1 | 1 | 5 |
|  | Sugar | Almost everyday | 5 | 1 | 5 |
|  |  | ≥1 time/week | 4 | 2 | 4 |
|  |  | ≥1 time/month | 3 | 3 | 3 |
|  |  | Occasionally | 2 | 4 | 2 |
|  |  | Rarely or never | 1 | 5 | 1 |
|  | Salt-preserved vegetable | Almost everyday | 5 | 1 | 5 |
|  |  | ≥1 time/week | 4 | 2 | 4 |
|  |  | ≥1 time/month | 3 | 3 | 3 |
|  |  | Occasionally | 2 | 4 | 2 |
|  |  | Rarely or never | 1 | 5 | 1 |
|  | Animal fat | Yes | 1 | 1 | 1 |
|  |  | No | 5 | 5 | 5 |
|  | Fish | Almost everyday | 1 | 1 | 1 |
|  |  | ≥1 time/week | 2 | 2 | 2 |
|  |  | ≥1 time/month | 3 | 3 | 3 |
|  |  | Occasionally | 4 | 4 | 4 |
|  |  | Rarely or never | 5 | 5 | 5 |
|  | Meat | Almost everyday | 1 | 1 | 1 |
|  |  | ≥1 time/week | 2 | 2 | 2 |
|  |  | ≥1 time/month | 3 | 3 | 3 |
|  |  | Occasionally | 4 | 4 | 4 |
|  |  | Rarely or never | 5 | 5 | 5 |
| Animal food | Egg | Almost everyday | 1 | 1 | 1 |
|  |  | ≥1 time/month | 3 | 3 | 3 |
|  |  | Occasionally | 4 | 4 | 4 |
|  |  | Rarely or never | 5 | 5 | 5 |
|  | Dairy products | Almost everyday | 1 | 1 | 1 |
|  |  | ≥1 time/week | 2 | 2 | 2 |
|  |  | ≥1 time/month | 3 | 3 | 3 |
|  |  | Occasionally | 4 | 4 | 4 |
|  |  | Rarely or never | 5 | 5 | 5 |
| Abbreviations: PDI: plant-based diet index; hPDI: healthy plant-based diet index; uPDI: unhealthy plant-based diet index. | | | | | |

| **Supplementary Table S2. Detalis of mediation models** | | | | |
| --- | --- | --- | --- | --- |
| Path | B | 95%CI | | p-Value |
|  |  | Lower | Upper |  |
| **PDI->Sleep quality->Depression** |  |  |  |  |
| Indirect effect | -0.031 | -0.044 | -0.020 | <0.001 |
| Direct effect | -0.020 | -0.057 | 0.020 | 0.274 |
| Total effect | -0.051 | -0.090 | -0.010 | 0.016 |
| Mediated proportion，n (%) | 61% | 28% | 196% | 0.016 |
| **uPDI->Sleep quality->Depression** |  |  |  |  |
| Indirect effect | 0.026 | 0.016 | 0.040 | <0.001 |
| Direct effect | 0.047 | 0.018 | 0.070 | 0.004 |
| Total effect | 0.072 | 0.042 | 0.100 | <0.001 |
| Mediated proportion，n (%) | 36% | 23% | 62% | <0.001 |
| **hPDI->Sleep quality->Depression** |  |  |  |  |
| Indirect effect | -0.023 | -0.039 | -0.010 | <0.001 |
| Direct effect | -0.068 | -0.105 | -0.030 | <0.001 |
| Total effect | -0.091 | -0.132 | -0.050 | <0.001 |
| Mediated proportion，n (%) | 25% | 10% | 47% | <0.001 |
| Abbreviations:PDI,plant-based diet index;uPDI,unhealthy plant-based diet index;hPDI,healthy plant-based diet index. | | | | |


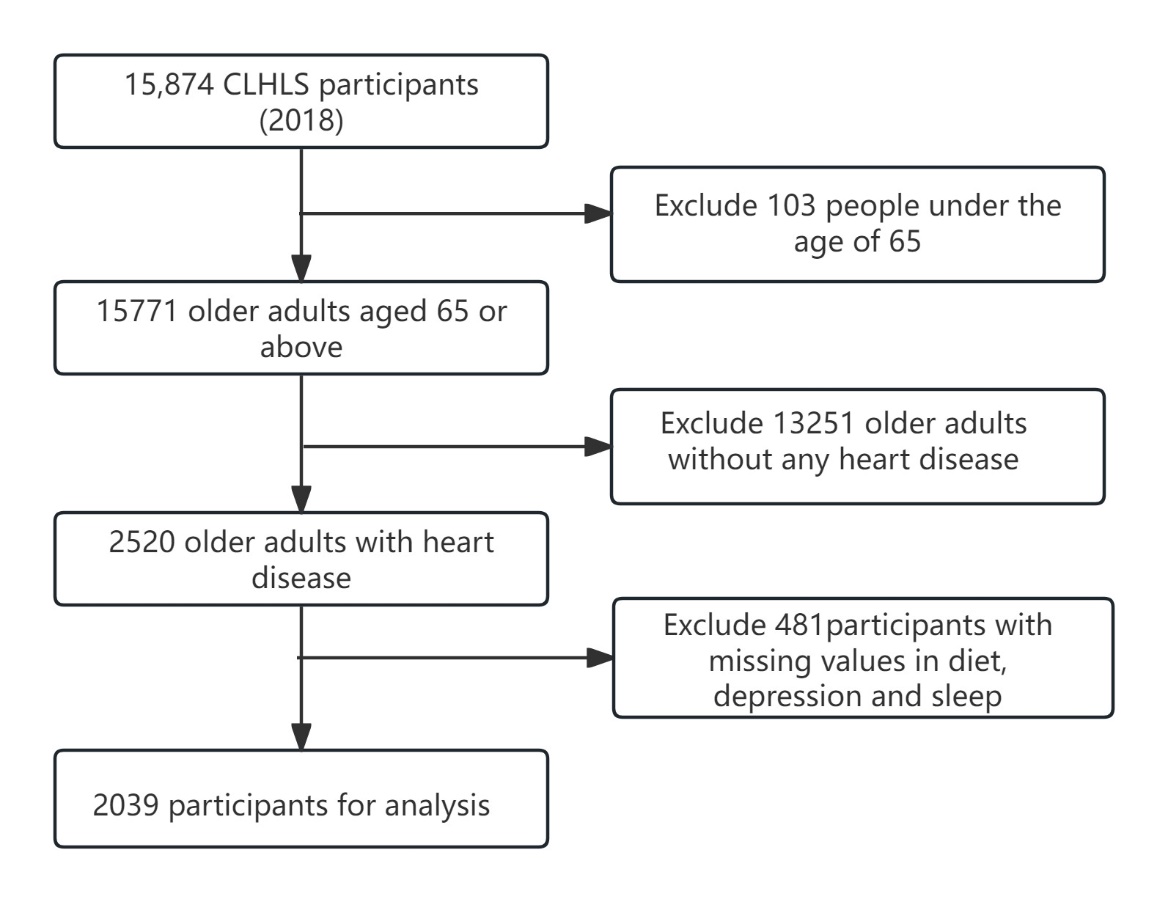


Figure S1 Flow chart for the screening of research subjects.


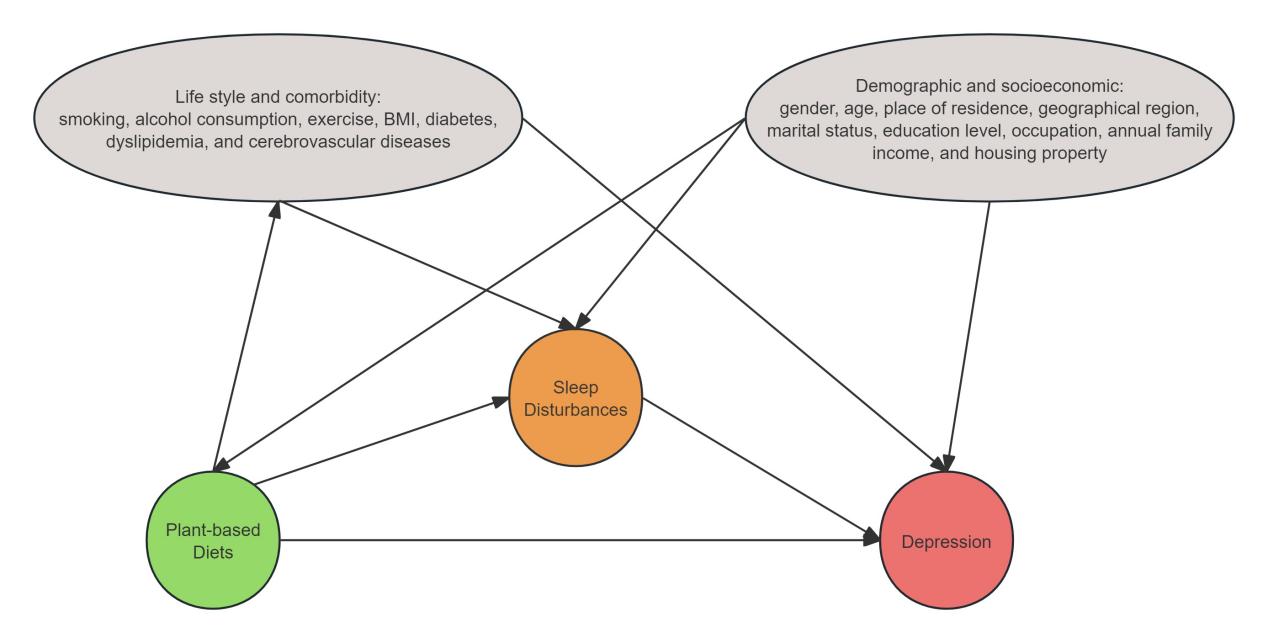


Figure S2 Directed Acyclic Graphs (DAG) of variables included.


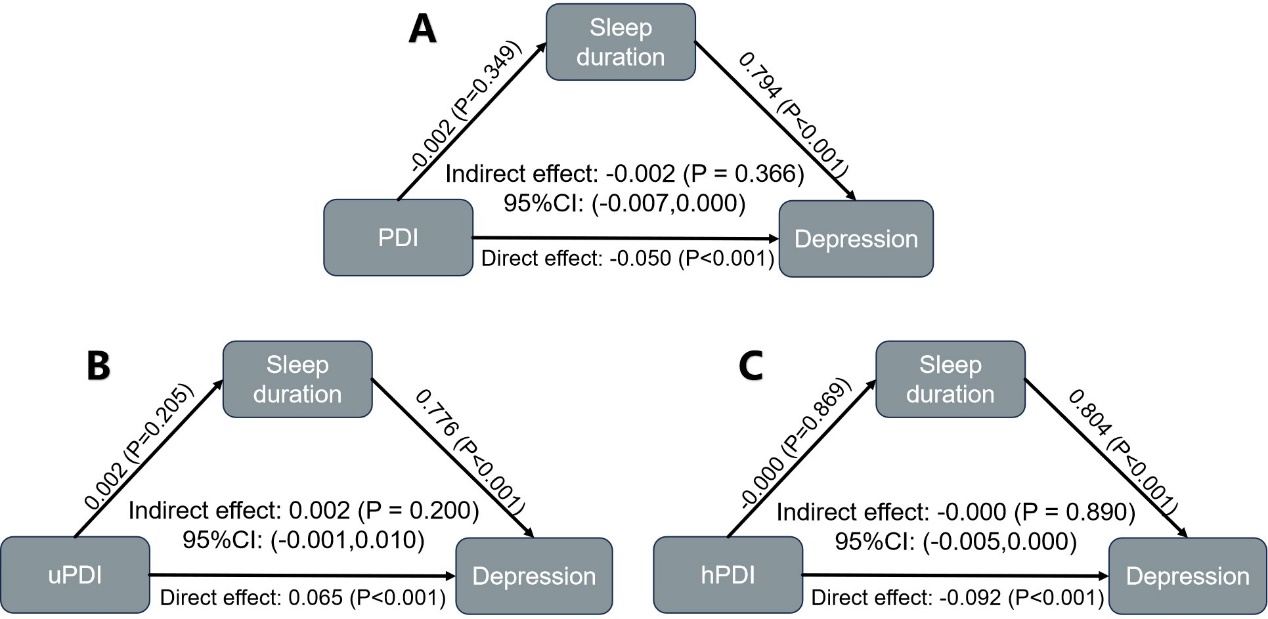


Figure S3 Results of the mediating effect of sleep duration.
